# *Tenuipalpus* Sensu Lato Donnadieu (Acari: Prostigmata: Tenuipalpidae); New Species Groups, a New Species, and Keys to the World Species †

**DOI:** 10.3390/ani13203278

**Published:** 2023-10-20

**Authors:** Nasreldeen Ahmed Elgoni, Muhammad Kamran, Fahad Jaber Alatawi

**Affiliations:** Department of Plant Protection, College of Food and Agriculture Sciences, King Saud University, Riyadh 11451, Saudi Arabia; nabdelkarim@ksu.edu.sa (N.A.E.); murafique@ksu.edu.sa (M.K.)

**Keywords:** opisthosomal setae, tropical regions, divisions, *Chamaerops* spp., related species, geographical distribution

## Abstract

**Simple Summary:**

Castro et al. divided the genus Tenuipalpus into two groups, i.e., Tenuipalpus sensu stricto and Tenuipalpus sensu lato. Four new species groups of the Tenuipalpus sensu lato group are proposed in this study, considering the total number of dorsal opisthosomal setae. Additionally, diagnostic keys to new species groups and the world species of Tenuipalpus sensu lato are developed.

**Abstract:**

Four new species groups of the *Tenuipalpus* sensu lato group are proposed in the present study based on the total number of dorsal opisthosomal setae, namely, *carolinensis* with ten pairs of setae (214 species), *dubinini* with nine pairs of setae (33 species), *granati* with eight pairs of setae (29 species), and *barticanus* with seven pairs of setae (7 species) Additionally, diagnostic keys to species groups and 273 species of the *Tenuipalpus* sensu lato are provided. Three species, *T*. *lustrabilis* Chaudhri, *T*. *guptai* Sadana and Gupta, and *T*. *solanensis* Sadana and Gupta, are synonymized with *T*. *punicae* Pritchard and Baker. One species, *T. rodionovi* Chalilova, is suggested as a junior synonym of *T*. *granati* Sayed, and eight species, *T*. *chiococcae* De Leon, *T*. *costarricensis* Salas and Ochoa, *T. ephedrae* Livschitz and Mitrofanov, *T*. *molinai* Evans, *T*. *santae* Manson, *T*. *simplychus* Cromroy, *T*. *tetrazygiae* De Leon, and *T*. *oxalis* (Flechtmann), belonging to the *carolinensis* species group, are not included in the key. Furthermore, a new species of *Tenuipalpus* sensu lato, *T*. *jazanensis* sp. nov., is described and illustrated based on females collected from the *Chamaerops* spp. (Arecaceae).

## 1. Introduction

*Tenuipalpus* Donnadieu is the largest genus in the family Tenuipalpidae Berlese (Acari: Prostigmata: Tetranychoidea) and consists of more than 320 species, distributed worldwide, especially in the tropical and subtropical regions [1,2,3]. The members of this genus are mostly oligophagous in their feeding habits [2]. The three species, i.e., *T*. *granati* Sayed, *T*. *punicae* Pritchard and Baker, and *T*. *eriophyoides* Baker, are serious pests of fruit trees worldwide [4].

The genus *Tenuipalpus* was erected by Donnadieu in 1875 [5]. Based on the number of dorsal setae and palp segmentation, Reck [6] and Mitrofanov [7] erected six genera by transferring some of *Tenuipalpus* species, *Extenuipalpus* Reck [6], *Aegyptopalpus* Mitrofanov [7], *Deleonipalpus* Mitrofanov [7], *Gnathopalpus* Mitrofanov [6], *Tuttlepalpus* Mitrofanov [7], and *Ultratenuipalpus* Mitrofanov [7]. Meyer [8] synonymized five of those genera with the genus *Tenuipalpus* by using the same morphological characters and proposed six species groups, namely, *albae*, *caudatus*, *elegans*, *granati*, *quadrisetosus*, and *trisetosus*. Later, Baker and Tuttle [9] recognized only two species groups, *caudatus* and *proteae*, based on the presence and absence of setae *f2* (seven and six pairs of dorsolateral setae), respectively. Further, they divided the *caudatus* group into three species subgroups based on the number of intercoxal setae (*3a* and *4a*). Meyer [10] followed the concept of Baker and Tuttle [9] and divided the *proteae* into three species subgroups and added two more species subgroups to the *caudatus* group.

Collyer [11] made the world key of 102 *Tenuipalpus* species. Additionally, some local keys were developed over time by Meyer [10], which included around 90 species from Africa; Baker and Tuttle [9] included 20 species from Mexico; Al-Gboory [12] included seven species from Iraq; Khanjani et al. [13] included nine species from Iran; Castro and Feres [14] included 12 species from Brazil; and Xu et al. [15] included 25 species from China.

For more than three decades, *Tenuipalpus* species groups, as proposed by Baker and Tuttle [9] and Meyer [10], were consistent. However, recently, Castro et al. [1] divided the genus into two groups by using a combination of characters: *Tenuipalpus* sensu stricto (36 species), with a pair of lateral projections associated with setae *c3* and only one pair of setae *4a*, and *Tenuipalpus* sensu lato (287 species), without a lateral projection associated with setae *c3* and one to four pairs of setae *4a*. Also, a world key to the species of *Tenuipalpus* sensu stricto was provided [1,16]. However, no diagnostic key to the world species of *Tenuipalpus* sensu lato has been developed yet.

The aims of the present study were to (i) classify all species of *Tenuipalpus* sensu lato into new species groups based on distinct morphological characters; (ii) develop diagnostic keys to species groups and 273 species of the *Tenuipalpus* sensu lato; and (iii) examine specimens of *Tenuipalpus* species collected from different regions of Saudi Arabia.

## 2. Materials and Methods

The published taxonomic literature of all known 287 species belonging to the *Tenuipalpus* sensu lato group was collected using different resources: the Acarology Laboratory King Saud University, Google Scholar, ResearchGate, and different acarological journals, and through personal communication (Dr. Qing Hai Fan, Fujian Agriculture and Forestry University, China; and Dr. Elizeu B. Castro, São Paulo State University, São José do Rio Preto campus, São Paulo, Brazil). Diagnostic keys to species groups and 273 species are provided. All specimens of the genus that were collected by the Acarology lab have been examined. The mounted specimens of *Tenuipalpus* species were examined and identified under a phase contrast microscope (DM2500, Leica, Wetzlar, Germany). Different mite body parts were pictured using an Auto-Montage software system v4.0.1.1 (Syncroscopy, Cambridge, UK) and drawn with Adobe Illustrator v27.7 (Adobe System Inc., San Jose, CA, USA). All measurements are in micrometers. The terminology used in the research follows that of Mesa et al. [2]. The specimens were deposited at the King Saud University Museum of Arthropods (KSMA, Acarology Section), Department of Plant Protection, College of Food and Agriculture Sciences, King Saud University, Riyadh, Saudi Arabia.

## 3. Results

The *Tenuipalpus* sensu lato group was divided into four species groups based on the total number of dorsal opisthosomal setae, namely, the *carolinensis* group—with ten pairs of setae (214 species), the *dubinini* group—with nine pairs of setae (33 species), the *granati* group—with eight pairs of setae (29 species), and the *barticanus* group—with seven pairs of setae (seven species). This proposed division did not consider whether a specific setae was absent or not (i.e., any setae among the opisthosomal setae can be absent). The diagnostic keys to those four new species groups and 273 species of *Tenuipalpus* sensu lato were also developed.

Among the *carolinensis* group, eight species were not included in the key: *T*. *chiococcae* De Leon, *T*. *costarricensis* Salas and Ochoa, *T. ephedrae* Livschitz and Mitrofanov, *T*. *molinai* Evans, *T*. *santae* Manson, *T*. *simplychus* Cromroy, *T*. *tetrazygiae* De Leon, and *T*. *oxalis* (Flechtmann) (Table 1). Three species; *T*. *guptai* Sadana and Gupta, *T*. *solanensis* Sadana and Gupta, and *T*. *lustrabilis* Chaudhri, were synonymized with *T*. *punicae* Pritchard and Baker. Two species, *T. simplex* Vitzthum and *T*. *jasmini* Khan were poorly described; they were tentatively placed in the new groups of *carolinensis* and *granati*, respectively. The species *T. rodionovi* Chalilova was suggested as a junior synonymy of *T*. *granati* Sayed.

**Figure 1:**
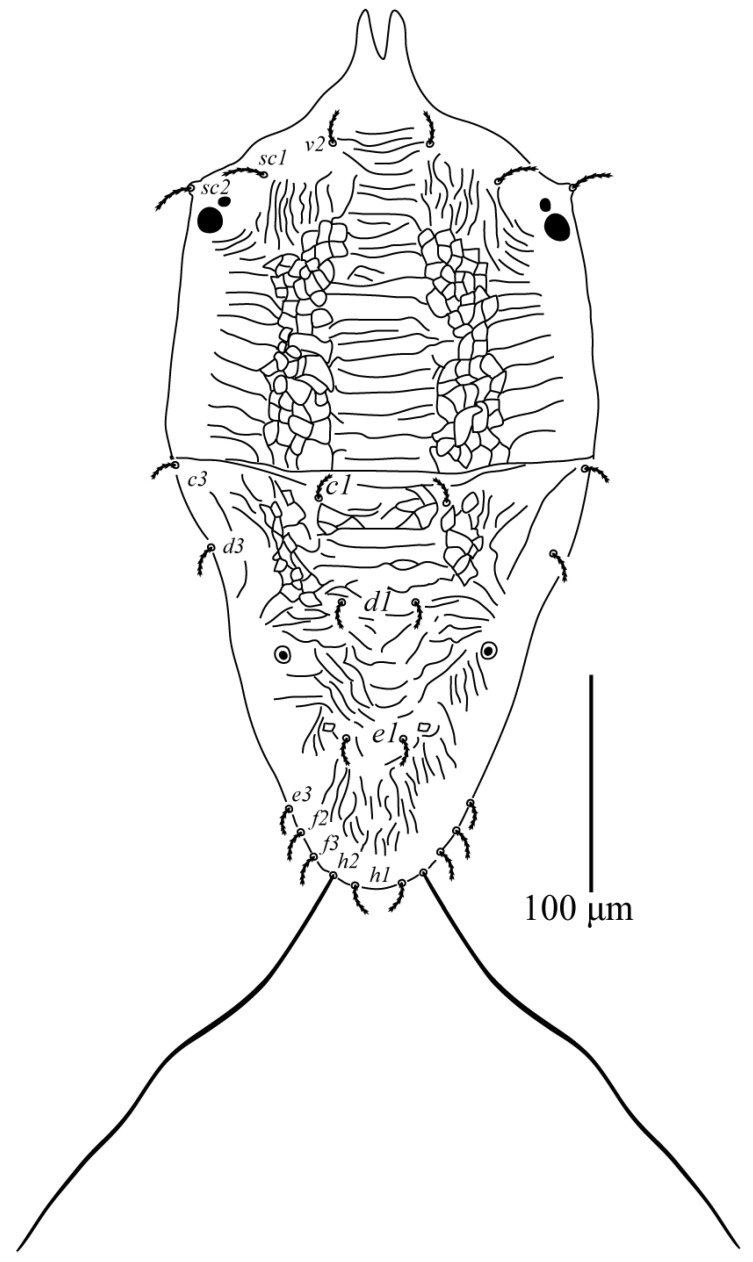
*Tenuipalpus jazanensis* sp.nov. Female. Dorsum. Scale bar: 100 µm.

**Figure 2:**
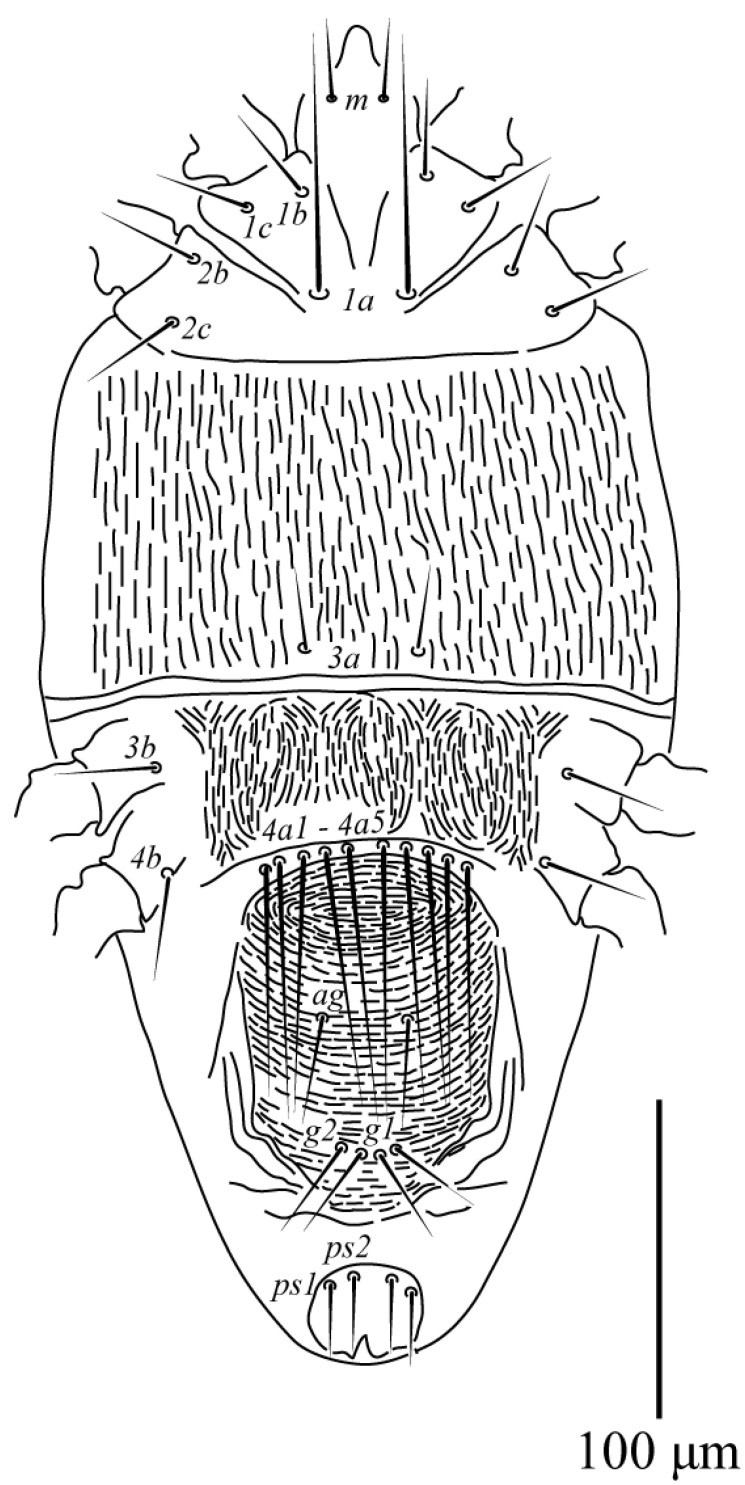
*Tenuipalpus jazanensis sp. nov.* Female. Venter. Scale bar: 100 µm.

**Figure 3:**
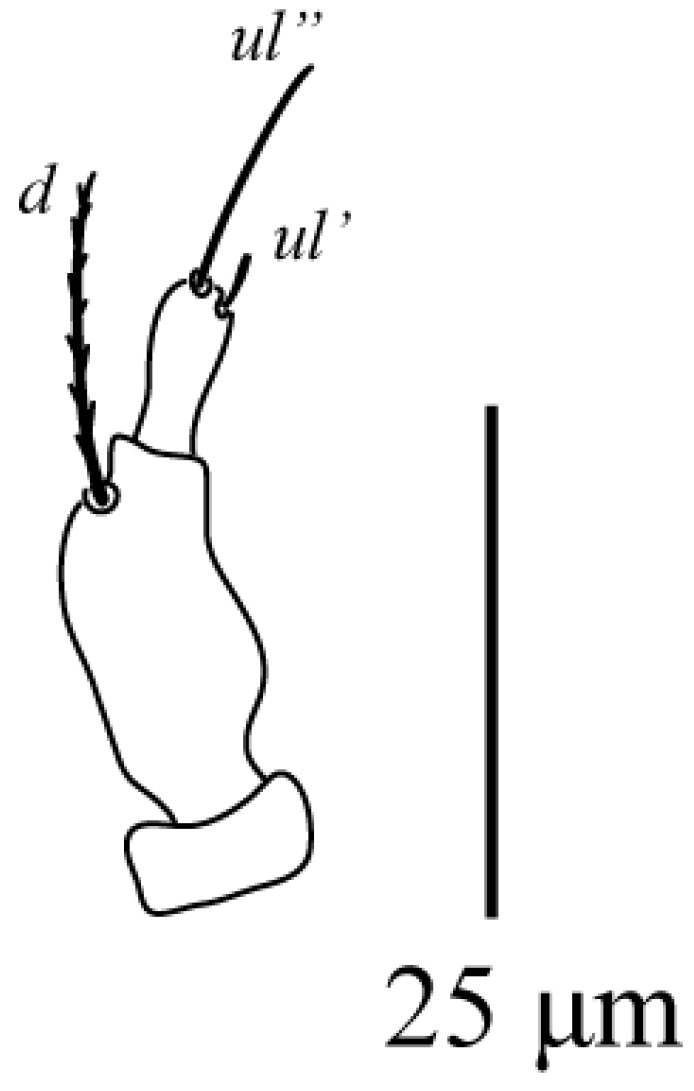
*Tenuipalpus jazanensis* sp. nov. Female. Palp. Scale bar: 25 µm.

**Figure 4:**
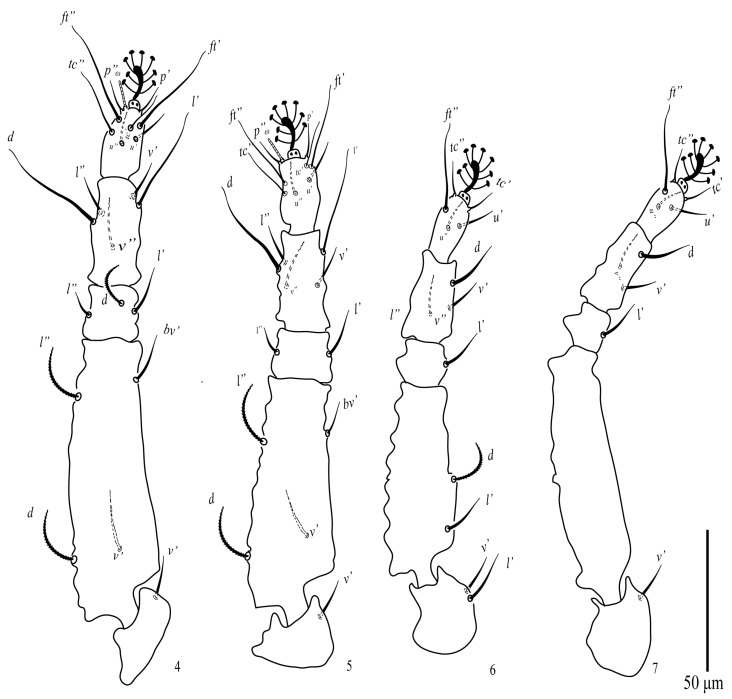
*Tenuipalpus jazanensis* sp. nov. Female. 4, Leg I; 5, leg II; 6, leg III; 7, leg IV. Scale bar: 50 µm.

Among the examined Tenuipalpus specimens collected from different regions of Saudi Arabia, a new species, *Tenuipalpus jazanensis* sp. nov., belonging to the Tenuipalpus sensu lato group, resulted. The new species is hereby fully described and illustrated based on females collected from the European fan palm, *Chamaerops* spp. (Arecaceae) from the Jazan region (Figure 1, Figure 2, Figure 3 and Figure 4).

### 3.1. Family Tenuipalpidae Berlese, 1913 [25]

Genus *Tenuipalpus* Donnadieu, 1875 [5].

**Type species:***Tenuipalpus palmatus* Donnadieu, 1875 [5] (=*Tenuipalpus caudatus* Dugès, 1834) [26].

**Diagnosis:** (modified after Castro et al. [16]): Prodorsum have three pairs of setae (*v*2, *sc1*, *sc2*) except the species *T. elegans* (Collyer) with two pairs of prodorsum setae (*sc1*, *sc2*), setae *v2* absent, opisthosoma with 7–10 pairs of setae; (*c3*, *d3*, *e3*, *f3*, *h1*, *h2* present; *c2*, *d2*, e2 absent; *c1*, *d1*, *e1*, *f2* present or absent (*d1*, *e1* rarely absent), setae *h2* elongate, flagellate; palp one- to three-segmented; venter with one to two pairs of setae *3a* (*3a1* always present; *3a2* present or absent) and one to five pairs of setae *4a* (*4a1* always present; *4a2*, *4a3*, *4a4, 4a5* present or absent); two pairs of pseudanal setae *ps1*-*2* (three pairs, *ps1*-*3*, rarely present).

### 3.2. Divisions of the Tenuipalpus Sensu Lato Group

*Tenuipalpus* sensu lato Castro et al. [1]

**Diagnosis:** (modified after Castro et al. [1]). Opisthosoma without one pair of lateral body projections associated with setae *c3*, venter with one to five pairs of intercoxal setae *4a*.

#### 3.2.1. *T. carolinensis* Species Group

**Diagnosis:** (based on female). Opisthosoma with ten pairs of dorsal setae. This group consists of 214 species.

#### 3.2.2. *T. dubinini* Species Group

**Diagnosis:** (based on female). Opisthosoma with nine pairs of dorsal setae. This group consists of 33 species.

#### 3.2.3. *T. granati* Species Group

**Diagnosis:** (based on female). Opisthosoma with eight pairs of dorsal setae. This group consists of 29 species.

#### 3.2.4. *T. barticanus* Species Group

**Diagnosis:** (based on female). Opisthosoma with seven pairs of dorsal setae. This group consists of seven species.

### 3.3. New Species

*Tenuipalpus* (sensu lato Castro et al. [1])

*T. carolinensis* group

*Tenuipalpus jazanensis* sp. nov.

urn:lsid:zoobank.org:act:ACDFDCC0-A7D3-4B3B-8618-27E9F14A6A44

**Diagnosis:** (based on Female). Propodosoma without lateral lobes anterior marginally; propodosoma with transverse striate medially and laterally, reticulated sublaterally. Hysterosoma medially with few reticulations in the area between setae *c1*-*c1* and sublaterally; one pair of seta *3a* and five pairs of setae *4a* (*4a1*−5) present; rostrum reaching to the middle of femur I; palp three-segmented; legs setal counts on coxae, 2–2–1–1 trochanters, 1–1–2–1; femora 4–4–2–0; genua 3–2–1–1; tibiae 5–5–3–3 and tarsus 8 (1)*–*8 (1)*–*5*–*5.

**Description Female (n = 5)**

Dorsum (Figure 1): Anterior margin of prodorsum deeply incised, depth of notch 25 (24–27), propodosoma without lateral lobes anterior marginally; propodosoma with transverse striate medially and laterally, reticulated sublaterally. Hysterosoma medially with few reticulations in the area between setae *c1* and sublaterally; dorsal body setae lanceolate serrate, almost equal in length, except setae *h2*; opisthosomal pores present; distance between setae *v2*–*h1* 339 (335–355), *sc2*–*sc2* 180 (160–240). Prodorsum slightly wider than proximal section of opisthosoma; anterior central of prodorsum with transverse striations; lateral regions with reticulations. All dorsal setae short (except *h2*), not more than 23 μm long; setae *v2*, *sc1*, and *sc2* serrate, *sc2* longer than *v2* and subequal in length with *sc1*; opisthonotum with reticulations; opisthosomal setae serrate; setae *e3* shorter than *f2* and *f3*, *h2* flagelliform and elongate serrate. Setal lengths: *v2* 16 (13–16), *sc1* 23 (20–23), *sc2* 23 (19–23), *c1* 13 (13–16), *c3* 13 (12–15), *d1* 12 (12–13), *d3* 14 (12–16), *e1* 13 (10–15), *e3* 12 (12–15), *f_2_* 14 (14–16), *f_3_* 14 (14–16), *h1* 14 (14–20), *h2* 233 (230–239). Distance between dorsal setae: *v2*–*v2* 43 (41–43), *sc1*–*sc1* 107 (95–107), *c1*–*c1* 57 (52–59), *c3*–*c3* 195 (192–197), *d1*–*d1* 32 (30–39), *d3*–*d3* 156 (160–169), *e1*–*e1* 25 (12–25), *e3*–*e3* 82 (82–90), *f2*–*f2* 71 (66–72), *f3*–*f3* 56 (52–57), *h2*–*h2* 41 (36–43), *h1*–*h1* 20 (20–23), *c1*–*d1* 45 (40–45), *d1*–*e1* 60 (60–62), *c1*–*c3* 66 (66–69), *d1*–*d3* 64 (59–65) *e1*–*e3* 41 (40–44), *c3*–*d3* 38 (30–38), *d3*–*e3* 121 (120–127), *e3*–*f3* 30 (28–36), *f3*–*f2* 10 (12–15), *h1*–*h2* 7 (7–10).

Venter (Figure 2): Ventral cuticle with broken longitudinal striations. Area posterior setae *4a* with broken transverse striate. All ventral setae smooth, setae *1a*, *4a* flagelliform, and elongate. Setal lengths: *1a* 82 (82–92), *1b* 30 (22–31), *1c* 30 (26–30), *2b* 33 (23–33), *2c* 33 (23–33), *3a* 25 (25–31), 3*b* 30 (21–31), *4a1* 90 (90–100), *4a*2 83 (82–91), *4a*3 83 (80–86), *4a4* 76 (76–84), *4a5* 73 (73–102), *4b* 33 (30–33), *ag* 36 (26–36), *g1* 33 (26–34), *g2* 33 (25–33), *ps1* 23 (18–25), *ps2* 23 (18–23). Distance between ventral setae: *1a*–*1a* 23 (19–24), *3a*–*3a* 30 (30–32), *4a1*–*4a1* 7 (5–7), *4a2*–*4a2* 21 (19–22), *4a3*–*4a3* 35 (31–36), *4a4*–*4a4* 49 (42–49), *4a5–4a5* 60 (57–62), *1a*–*3a* 108 (102–110), *3a*–*4a1* 57 (57–60), *1b*–*1c* 14 (13–19), *2b*–*2c* 20 (19–25).

Gnathosoma (Figure 3): Ventral setae *m* 25 (14–25); distance between setae *m*–*m* 15 (10–15) Palps 3-segmented (Figure 3), median segment elongates, bearing one barbed seta *d* 15 (12–20); distal segment short, with eupathidium *ul*′ 4 (4–7) *ul*″ 15 (12–15).

Legs (Figure 4): Setae on legs as follows: coxa 2–2–1–1; trochanters 1–1–2–1; femora 4–4–2–0; genu 3–2–1–1; tibiae 5–5–3–3; tarsus 8 (1ω)*–*8 (1ω)*–*5*–*5; femur IV without seta. Leg I–IV setal count as follows (solenidia in parenthesis).

**Males and immature.** Unknown

**Type Materials.** Holotype female, four paratype females, from *Chamaerops* sp. (Arecaceae) Wadi Baydh, Jazan, 17°37.559’ N, 42°22.196’ E, 10 October 2020, coll. J. H. Mirza, H.M.S. Mushtaq and E.M. Khan.

**Etymology:** The specific epithet (*jazanensis*) is derived from the type region, Jazan.

**Remarks:** The new species, *Tenuipalpus jazanensis* sp. nov., belongs to the *carolinensis* species group. This species group is distinguished from other species groups of *Tenuipalpus* sensu lato by having ten pairs of opisthosomal setae. *Tenuipalpus jazanensis* sp. nov resembles *T*. *pareriophyoides* Meyer and Gerson and *T*. *eriophyoides* Baker by having more than three pairs of intercoxal setae *4a*, one pair of setae *3a*, dorsal setae lanceolate serrate and dorsum with irregular striate laterally. The new species differs from *T*. *pareriophyoides* and *T*. *eriophyoides* by the following characters; prodorsum medially with transverse striations; area in between setae *c1*, *d1* and *d3* with few reticulations (prodorsum medial area mostly smooth or punctate; area in between setae *c1*, *d1* and *d3* with rugose patten or striate); genu I with three setae (genu I with two setae); setae *g* and *ag* smooth (setae *g* and *ag* serrate in *T*. *pareriophyoides*; setae *g* and *ag* serrate in *T*. *eriophyoides*).

### 3.4. Key to the Groups and Species Groups of Tenuipalpus (Based on Females)

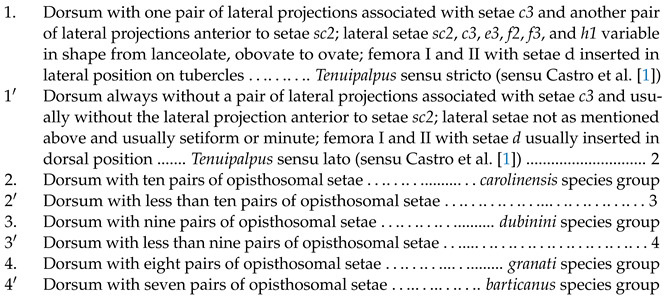



### 3.5. Key to the World Species of the T. carolinensis Species Group (Based on Females)

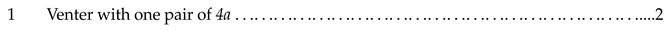

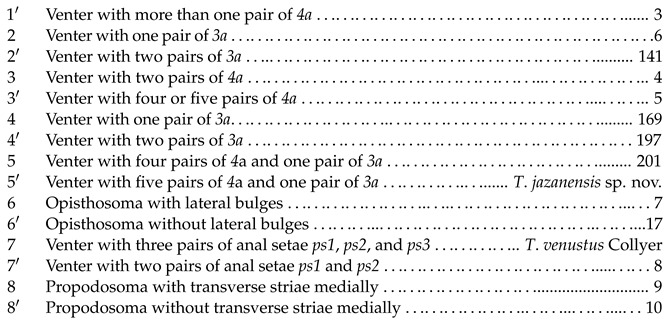

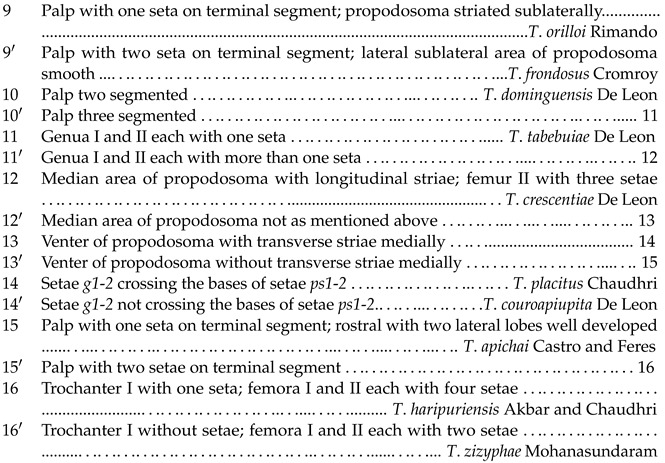

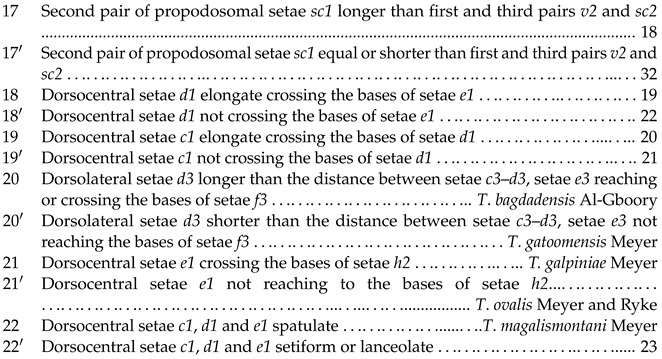

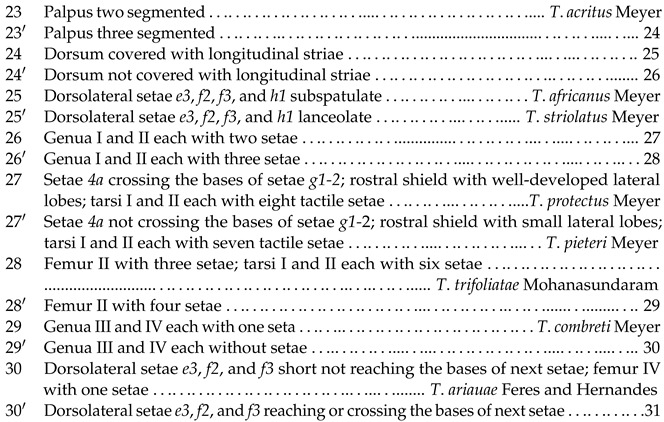

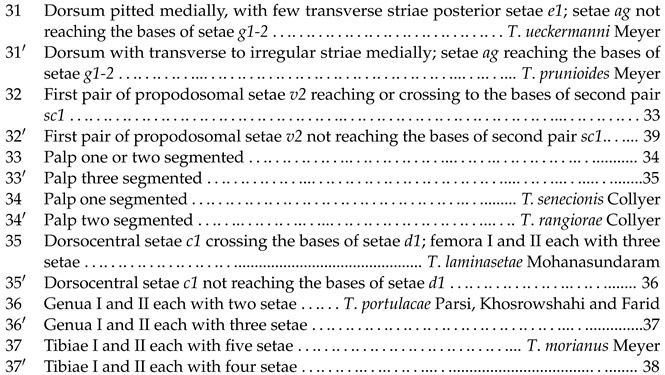

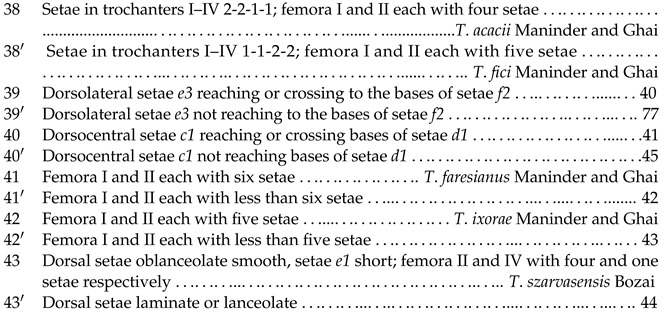

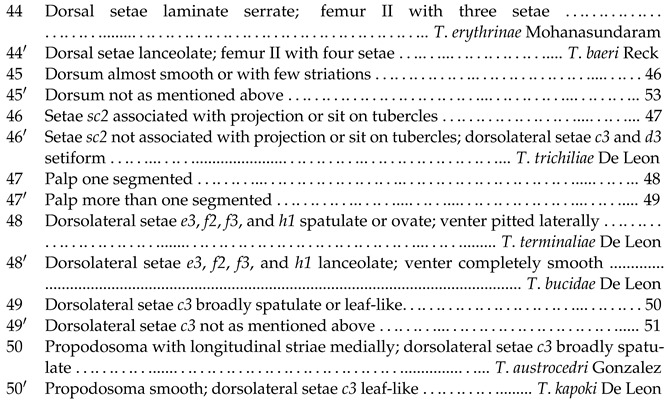

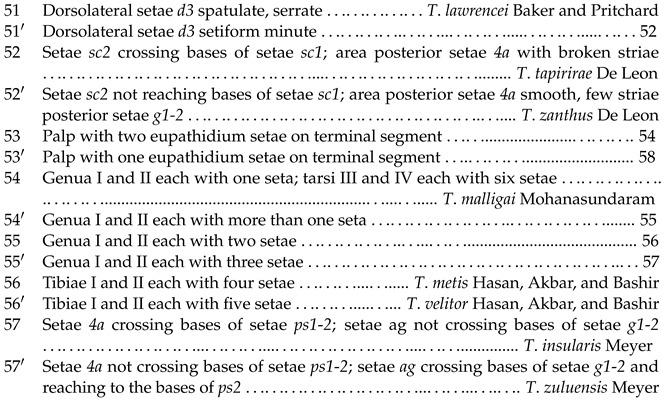

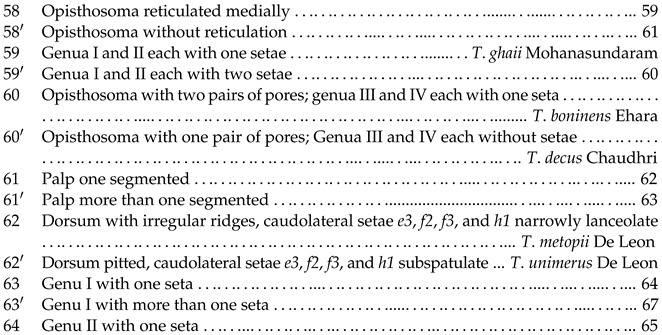

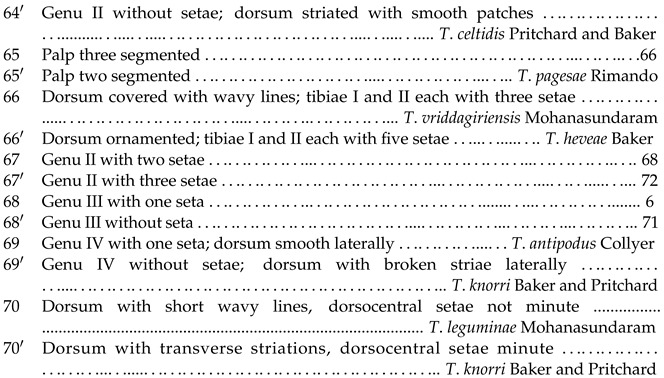

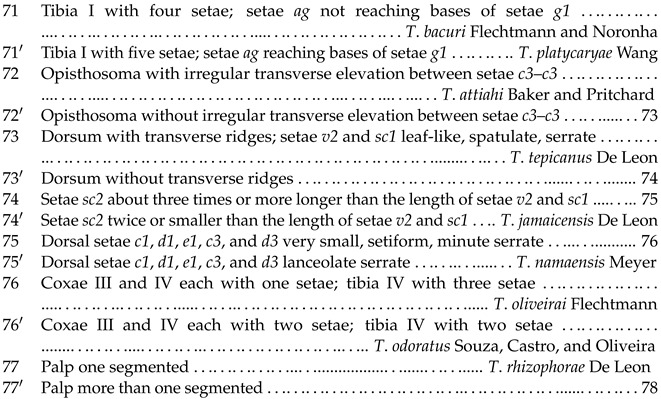

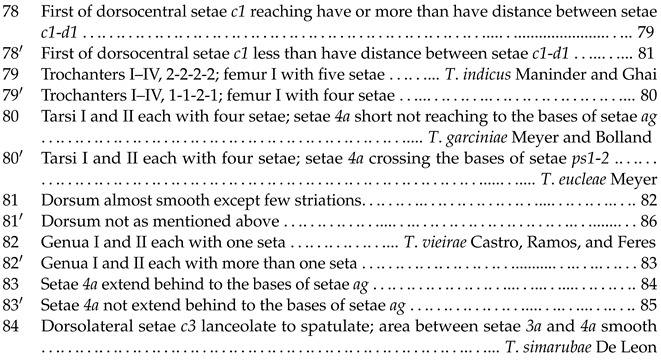

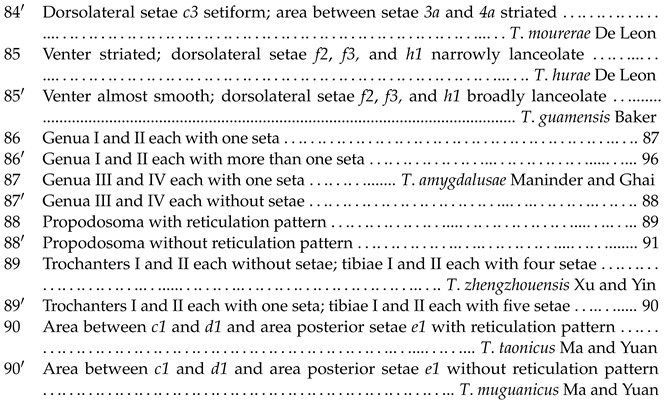

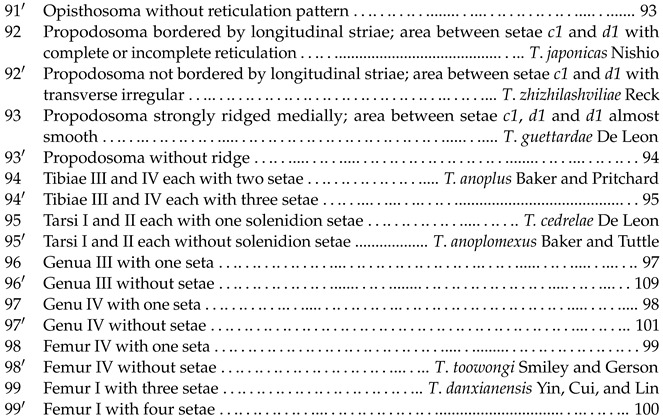

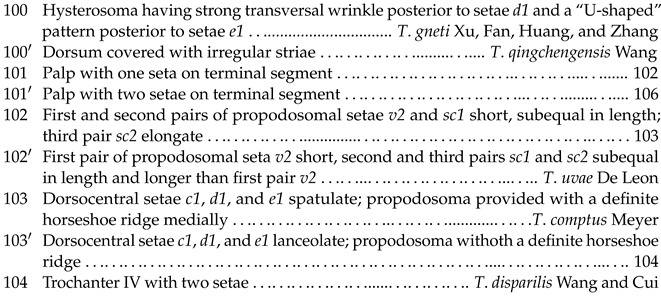

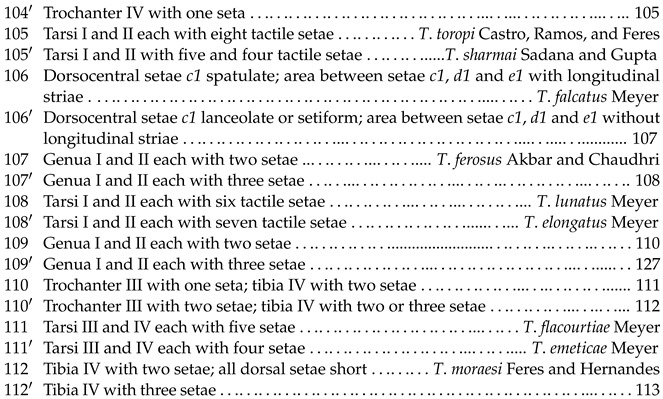

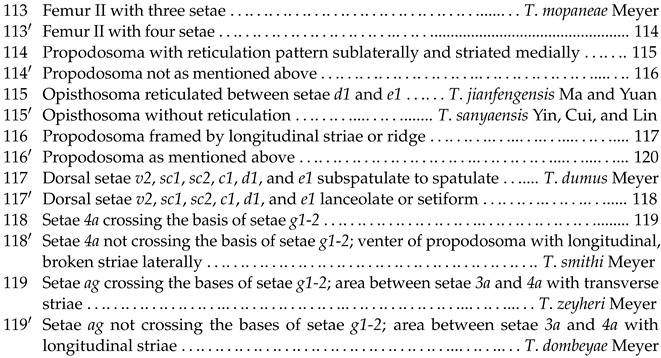

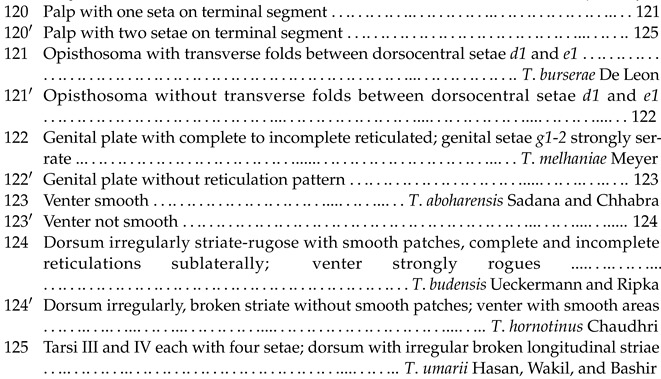

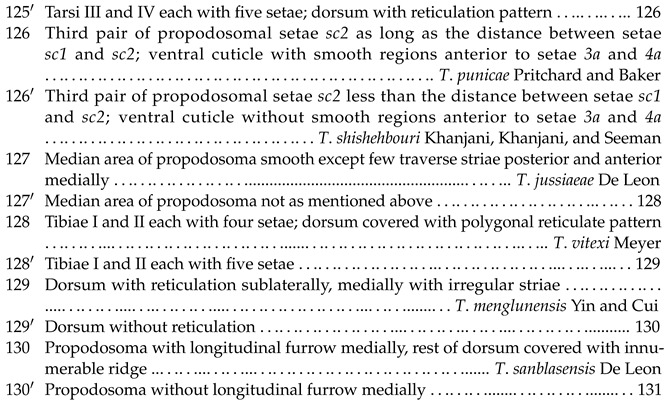

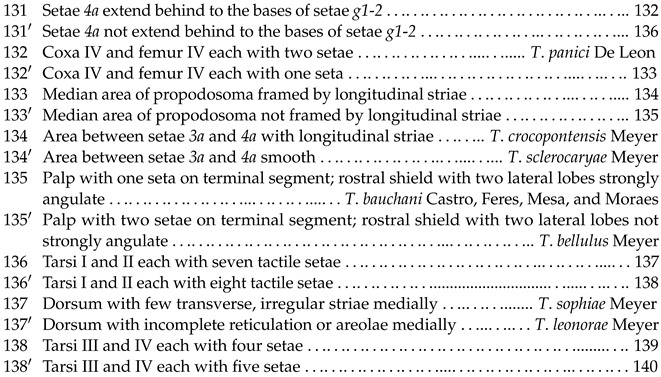

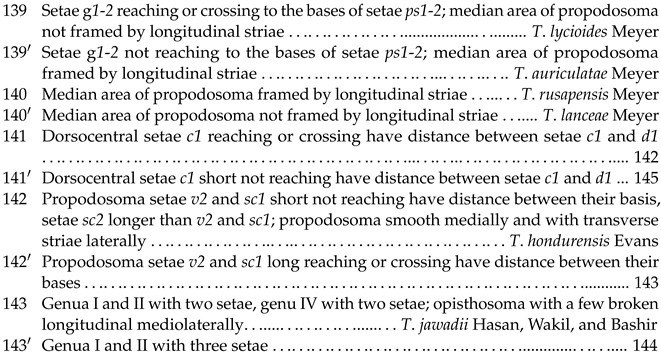

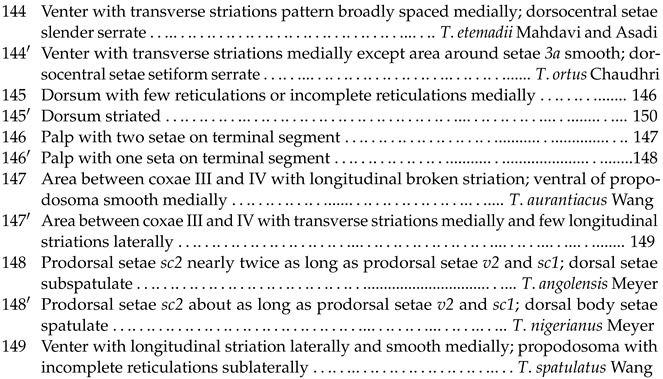

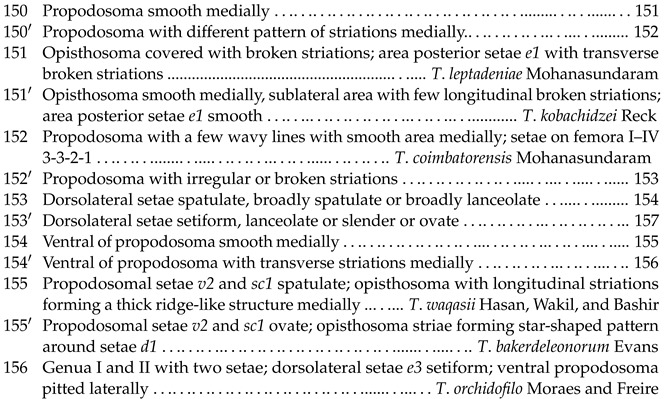

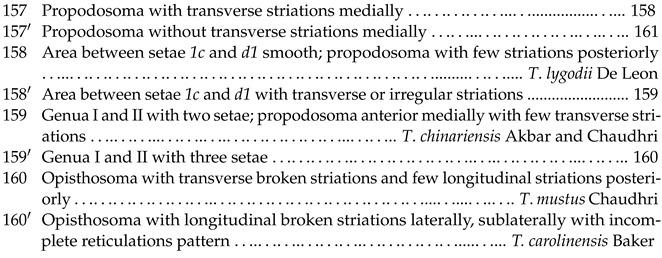

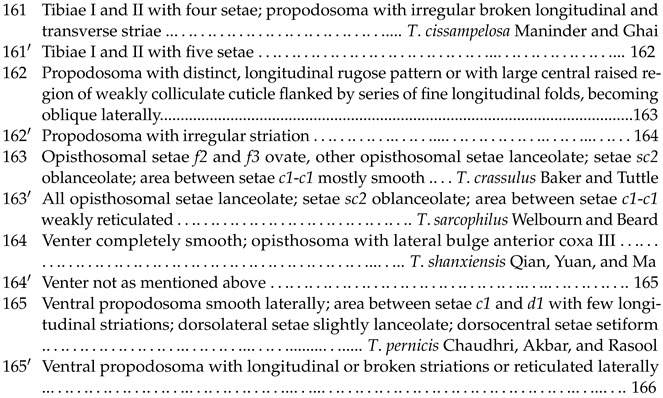

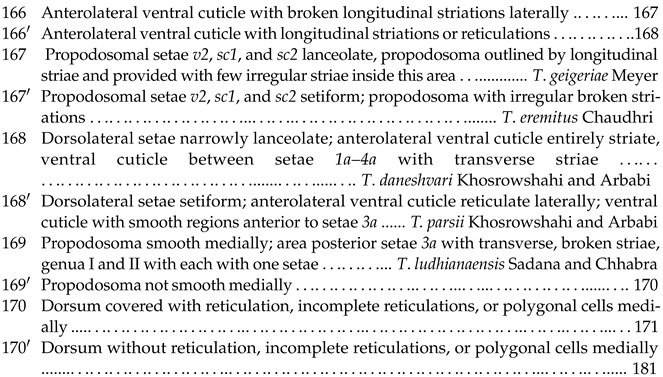

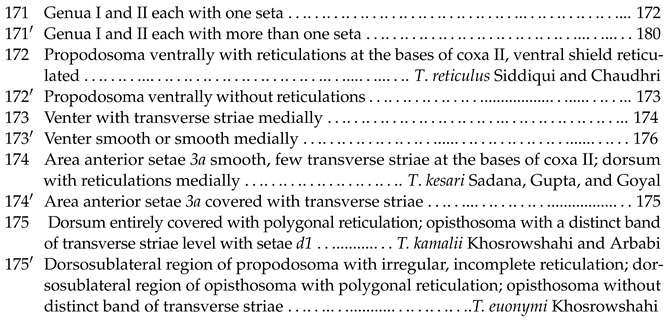

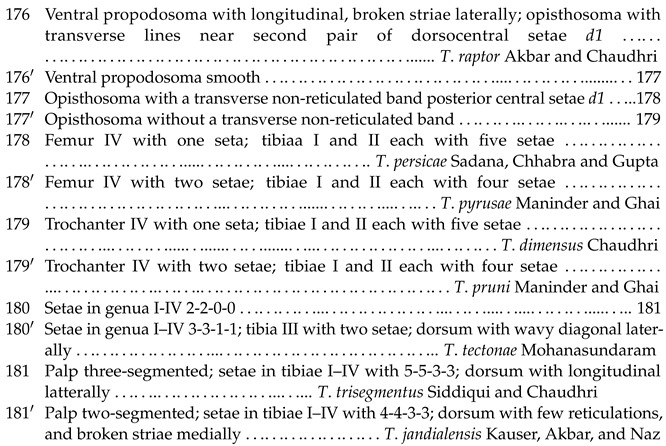

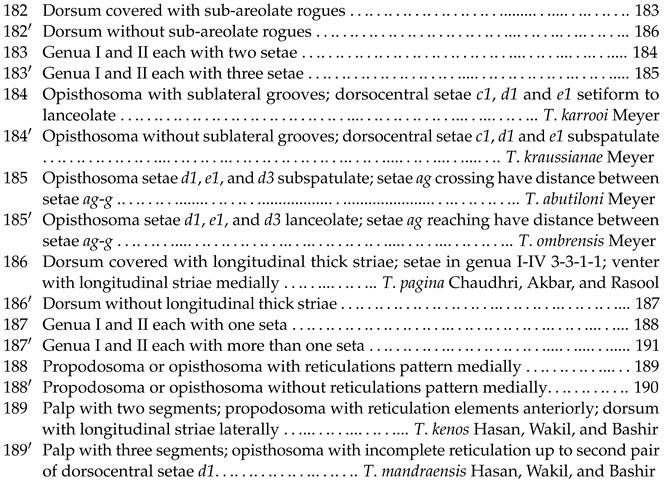

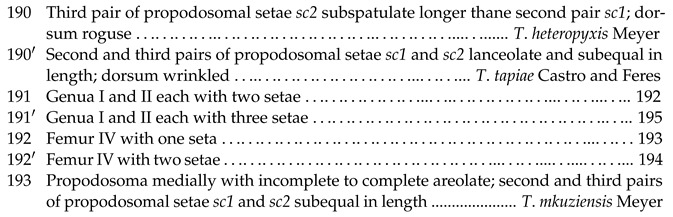

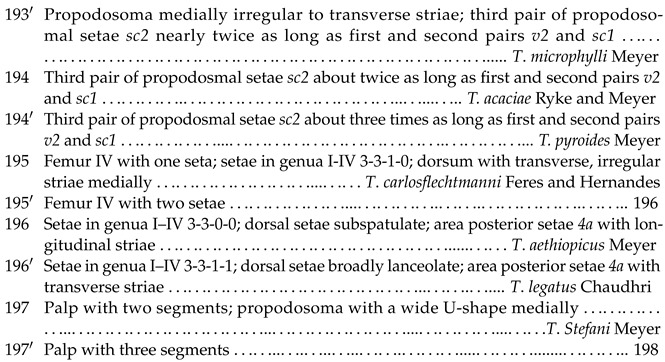

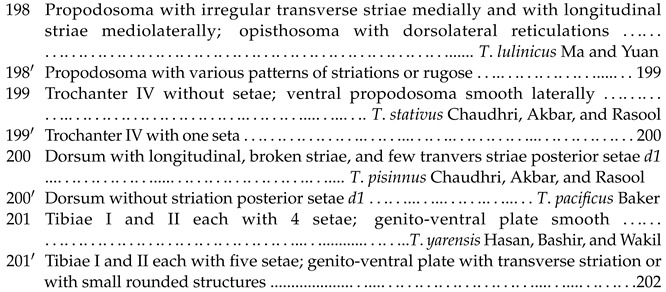

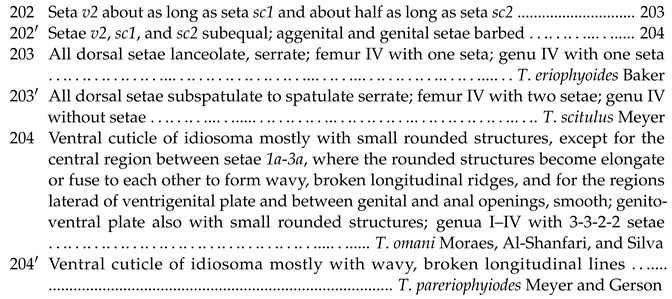





































































### 3.6. Key to the World Species of the T. dubinini Species Group (Based on Females)

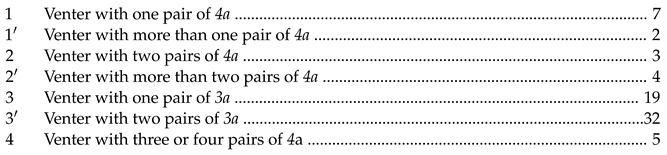

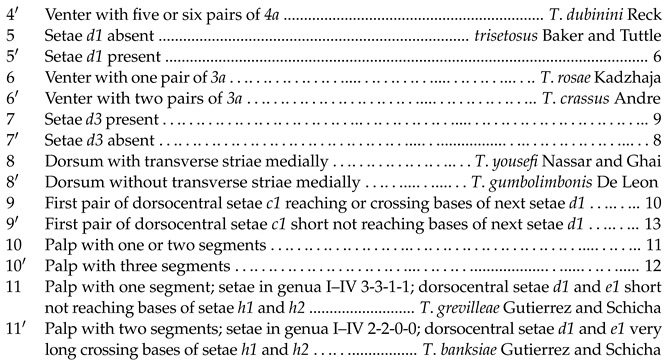

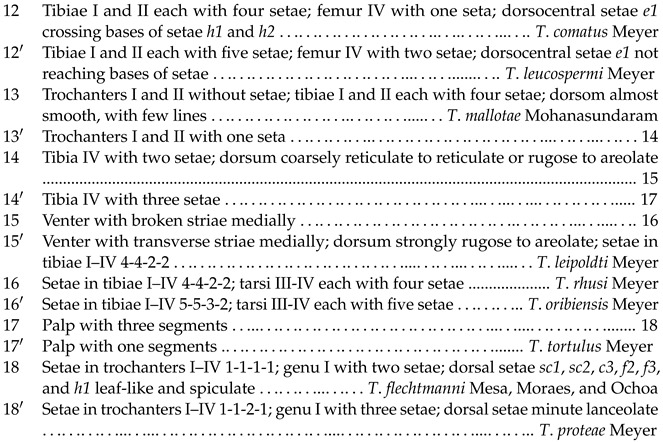

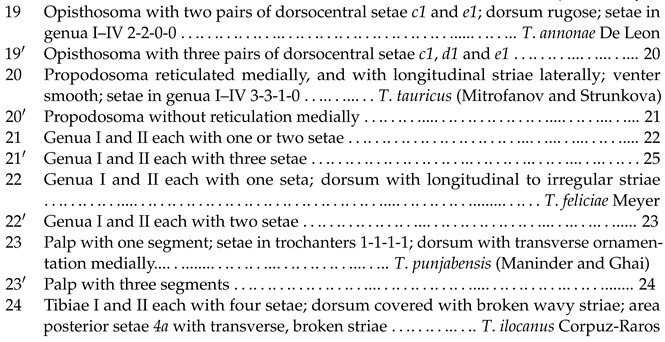

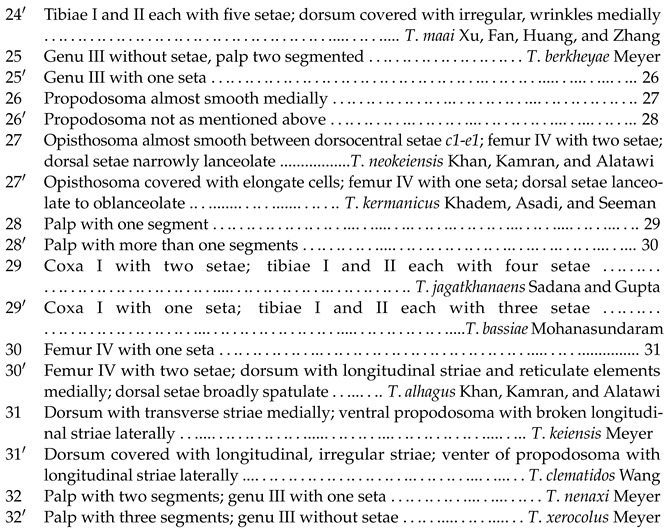











### 3.7. Key to the World Species of the T. granati Species Group (Based on Females)

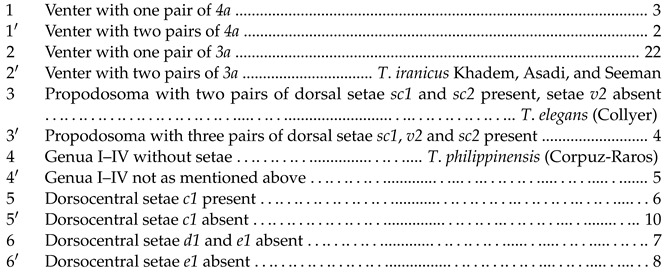

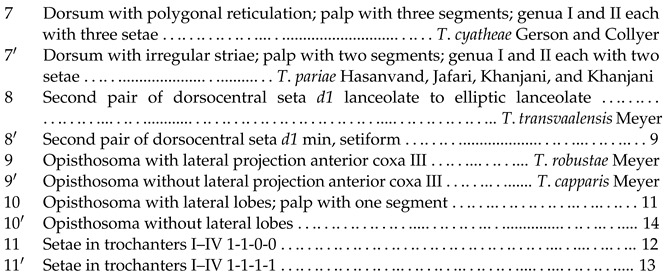

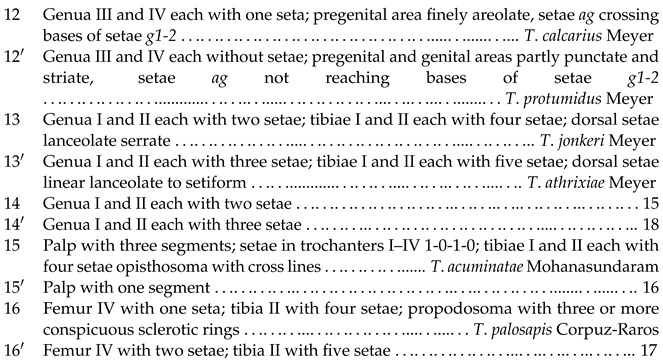

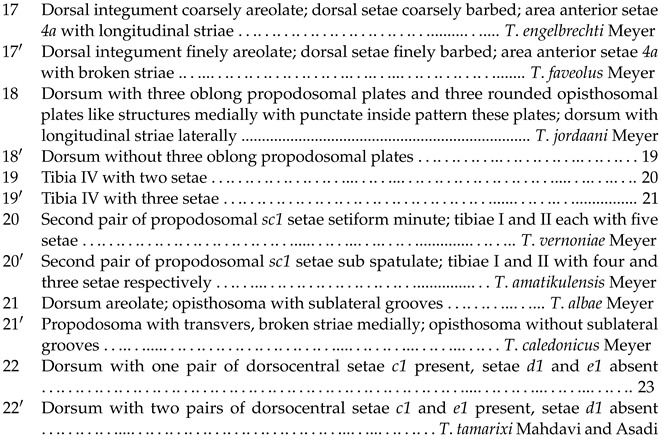

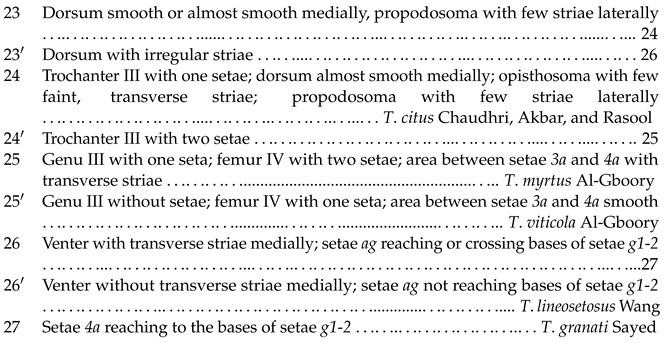

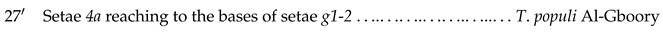













### 3.8. Key to the World Species of the T. barticanus Species Group (Based on Females)

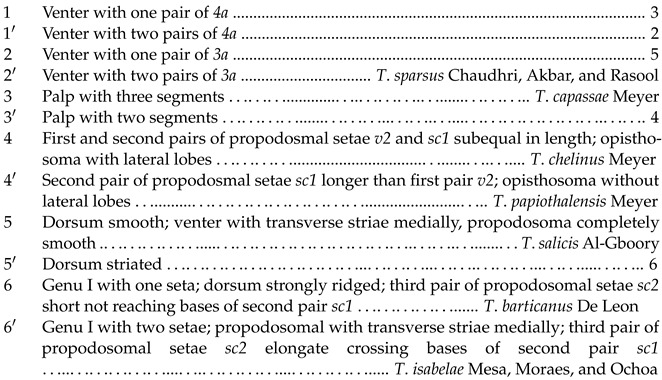



## 4. Discussion

The number of setae (dorsocentral and dorsolateral setae) has been used by different authors to erect tenuipalpid genera by transferring species from the genus *Tenuipalpus*, e.g., *Aegyptopalpus* Mitrofanov [7], *Deleonipalpus* Mirofanov [7], *Gnathopalpus* Mitrofanov [7], and *Tuttlepalpus* Mitrofanov [7]. Later, some of those genera were synonymized with the genus *Tenuipalpus* [8].

Different species groups were proposed based on the number of dorsocentral setae or dorsolateral setae [8,9,10]. According to the number of dorsolateral setae, two species groups, *caudatus* and *proteae*, based on the presence and absence of setae *f2* (with seven and six pairs of dorsolateral setae), respectively. Later, these two species groups were divided into species subgroups based on the number intercoxal setae *3a* and *4a* were recognized [9,10]. Also, the character of dorsocentral and dorsolateral setae have been used as a first couplet in the different keys to classify the *Tenuipalpus* species (Al-Gboory [12]; Khanjani et al. [13]; Castro et al. [16]; Xu et al. [15]). The literature of 287 species of *Tenuipalpus* sensu lato signified the importance of dorsal opisthosomal setae as a prominent diagnostic morphological character. However, using the presence and absence of dorsolateral setae *f2* (seven and six pairs of setae, respectively) could not be helpful, because some species (i.e., *T*. *clematidos* Wang, *T*. *flechtmanni* Mesa, Moraes, and Ochoa, *T*. *isabelae* Mesa, Moraes, and Ochoa, and *T. salicis* Al-Gboory) have six of dorsolateral setae (*f2* present), but the dorsolateral setae (*d3*) is absent. Hence, those species can not be placed in any species groups that proposed Baker and Tuttle [9] and Meyer [10] because of these grouping *caudatus* and *proteae* is based on presence and absense of setae *f2*. However, some previous works are considering the number of dorsolateral setae to distinguish these two species groups, regardless of which dorsolateral seta is absent. For example, Mahdavi and Asadi [27] included two species (*T*. *clematidos* Wang and *T. salicis* Al-Gboory) in the *proteae* species group, which have setae *f2* present. Therefore, we found that using the total number of opisthosomal setae is proper for dividing species groups, while the absence or presence of certain opisthosomal setae may be used for species differentiation in diagnostic keys. Hence, in this study, the four new species groups were proposed based on the number of opisthosomal setae.

### 4.1. Further Notes on the Poorly Described and Illustrated Species of the Species Group Carolinensis

The following remarks and additional notes are about those eight species belonging to the species group *carolinensis*, which were not included in the key due to poor/incomplete descriptions and illustrations. Their related species and a possible place in the diagnostic key are provided as follows:

**Tenuipalpus chiococcae De Leon**

The species *T*. *chiococcae* was originally described as close to *T*. *pigrus* Pritchard and Baker [17]. The recent classification by Castro et al. [1] placed *T*. *pigrus* in *Tenuipalpus* sensu stricto since this species bears a pair of lateral body projections associated with setae *c3*. The result of our study placed *T*. *chiococcae* among five species in the diagnostic key, i.e., *T*. *aurantiacus* Wang, *T*. *angolensis* Meyer, *T*. *nigerianus* Meyer, *T*. *spatulatus* Wang, and *T*. *obvelatus* Wan.

**Tenuipalpus costarricensis Salas and Ochoa**

Previously, *T*. *costarricencis* was considered close to *T*. *granati* Sayed [18]. However, in our study *T*. *granati* is placed in the group *granati*, while *T*. *costarricensis* came closer to three species; *T*. *eucleae* Meyer, *T*. *garciniae* Meyer and Bolland, and *T*. *indicus* Maninder and Ghai in the species group *carolinensis*.

**Tenuipalpus ephedrae Livschitz and Mitrofanov**

For the species *T. ephedrae*, the morphological characters were obtained from a poor redescription [19], and the original description was not found. The taxonomic information available in the redescription only helped to place this species in the group *carolinensis*.

**Tenuipalpus molinai Evans**

The species *T*. *molinai* was originally described as close to *T*. *pedrus* Manson [20]. The closely related *T*. *pedrus* Manson had been transferred to the genus *Colopalpus* Pritchard and Baker, previously [28]. However, the available morphological characters indicate that the species *T*. *molinai* belongs only to the species group *carolinensis*, but could not be assigned in the key.

**Tenuipalpus oxalis Flechtmann**

The species *T*. *oxalis* is poorly described and illustrated, and the related species was not provided [21]. The available morphological characters helped to designate the species only to the level of the species group *carolinensis*.

**Tenuipalpus santae Manson**

The species *T*. *santae* is morphologically close to *T*. *celtidis* Pritchard and Baker in the original description [22]. *Tenuipalpus santae* could not be placed due to missing leg cheatotaxy and other diagnostic characters.

**Tenuipalpus simplychus Cromroy**

The species *T*. *simplychus* was described as closely related to *T*. *knorri* Baker and Pritchard in the original description [23]. Due to missing leg chaetotaxy information, *T*. *simplychus* could not be assigned a certain place in the diagnostic key.

**Tenuipalpus tetrazygiae De Leon**

The species *T*. *tetrazygiae*, it was distinguished by the author from other described species of that time by the shape of dorsocentral setae and dorsum covered with irregular ridges [17]. Although this species has been placed in the *caroliensis* group, no certain place could be identified in the diagnostic key.

### 4.2. Synonymy of Some Species of the Carolinensis Species Group

The species *T*. *lustrabilis* was previously reported as a suspected junior synonym of *T*. *punicae* Pritchard and Baker [2,13]. We reviewed the original description and illustration [29] as well as the characters of *T*. *lustrabilis* in the published key by Meyer [8]. *T*. *lustrabilis* is hereby synonymized with *T*. *punicae* based on the number of shared characteristics, i.e., leg chaetotaxy, palp segmentation, shape and number of dorsal setae, pattern of dorsal reticulations, as well as its geographic distribution (Pakistan) and host plant (*Punica granatum*).

Similarly, the species *T*. *guptai* Sadana and Gupta was also suggested as a junior synonym of *T*. *solanensis* Sadana and Gupta [2,13]. We found that both species share most of their morphological features except for the number of setae on tarsi I-II (5-5 in *T*. *guptai* vs 7-7 in *T*. *solanensis*). This character has been commented to be a miscalculation [13], especially that *T*. *guptai* was described based on a single holotype female while *T*. *solanensis* only from three females. Moreover, both species were described based on specimens collected from the same host plant (*P*. *granatum*), the same type locality (India), on the same collection date (22-VI-1981), and have been mounted on the same slide (slide#91) [30].

Interestingly, the related species of *T*. *solanesis* is *T*. *lustrabilis*, which is declared as junior synonym of *T*. *punicae*. A detailed comparison of available description for both of these species show they are very similar; sharing leg chaetotaxy, palp segmentation, shape and number dorsal setae, pattern of dorsal reticulations, and host plant. Hence, the two species (*T*. *guptai* and *T*. *solanensis*) are also synonymized with *T*. *punicae*.

*Tenuipalpus rodionovi* Chalilova was described poorly without illustrations [31]. Therefore, it is neither assigned to any of the four species groups and not placed in the diagnostic keys. However, it was mentioned in the original description that it resembles three species: *T*. *granati* Sayed, *T*. *zhizhilashviliac* Reck, and *T*. *kobachidzei* Reck. The latter two species belong to the species group *carolinensis*, while the former one belongs to the *granati* new species group. Pritchard and Baker [32] and Wainstein [33] suspected this species as a junior synonym of *T*. *granati*. There is a need to check the type specimens of this species to validate its status. Hence, *T*. *rodionovi* is considered as a suggested synonym of *T*. *granati* until further studies are made.

## 5. Conclusions

The history of the genus *Tenuipalpus* is complicated due to taxonomic and classification-based modifications. This research indicates that the genus *Tenuipalpus* needs more taxonomical studies to raise the level of groups and species groups to higher taxonomical ranking, by using persistent and strong morphological characters. This may even direct future research in the family Tenuipalpidae to study the other closely related genera, in order to validate their status.

## Figures and Tables

**Table 1:** List of species not included in the diagnostic key of the new species group *carolinensis*.

Species (Geographic Distribtuion)	Host Plant	Related Species (Geographical Distribution)	Host Plant
*T*. *chiococcae* [17] **(USA)**	*Chiococca pinetorum*	*T*. *pigrus* Pritchard and Baker (USA)	*Umbellularia californica*
*T*. *costarricensis* [18] **(Costa Rica)**	*Cedrela* sp.	*T*. *granati* Sayed (Worldwide)	Polyphagous
*T. ephedrae* [19] **(Ukraine)**	*Ephedra distachya*	-	-
*T*. *molinai* [20] **(Honduras)**	Asteraceae: Unidentified plant	-	-
*T*. *oxalis* [21] **(Brazil)**	*Oxalis* sp.	-	-
*T*. *santae* [22] **(Costa Rica)**	Unidentified (fence tree)	*T*. *celtidis* Pritchard and Baker (USA)	*Celtis* sp.
*T*. *simplychus* [23] **(Puerto Rico)**	*Cordia sulcata*	*T*. *knorri* Baker and Pritchard (Argentina)	-
*T*. *tetrazygiae* [17,24] **(India and USA)**	*Anacardium occidentale* and *Etrazygia bicolor*	-	-

## Data Availability

All the relevant data has been provided here in the manuscript.
